# *fac*-Aqua­dichloridotris(tetra­methyl­ene sulfoxide-κ*S*)ruthenium(II)

**DOI:** 10.1107/S1600536809000439

**Published:** 2009-01-10

**Authors:** Radhey S. Srivastava, Carlos F. Gonzales, Frank R. Fronczek

**Affiliations:** a Department of Chemistry, University of Louisiana at Lafayette, Lafayette, LA 70504, USA; b Department of Chemistry, Louisiana State University, Baton Rouge, LA 70803-1804, USA

## Abstract

The title mol­ecule, [RuCl_2_(C_4_H_8_OS)_3_(H_2_O)], is the isomer with the two chloride ligands *cis* and the three *S*-coordinated tetra­methyl­ene sulfoxide ligands facial relative to the Ru(II) center. The Ru—Cl distances are 2.4161 (7) and 2.4317 (7) Å, the Ru—O distance is 2.1540 (19) Å, and the Ru—S distances are in the range 2.2254 (8)–2.2657 (7) Å, with the shortest being that *trans* to the aqua ligand. The coordinated water mol­ecule forms inter­molecular hydrogen bonds with Cl and sulfoxide O atoms.

## Related literature

For background literature, see: Aldinucci *et al.* (2007 ▶). For related structures, see: Srivastava & Fronczek (2003 ▶); Srivastava *et al.* (2004 ▶); Allen (2002 ▶). For hydrogen-bonding patterns, see: Etter (1990 ▶).
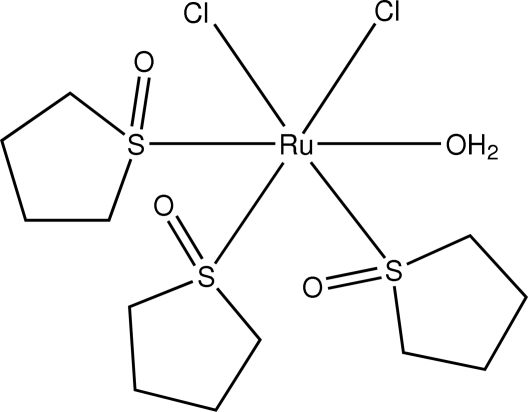


## Experimental

### 

#### Crystal data

[RuCl_2_(C_4_H_8_OS)_3_(H_2_O)]
*M* *_r_* = 502.48
Monoclinic, 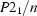

*a* = 14.302 (3) Å
*b* = 7.7877 (15) Å
*c* = 17.248 (3) Å
β = 109.917 (9)°
*V* = 1806.2 (6) Å^3^
*Z* = 4
Mo *K*α radiation
μ = 1.52 mm^−1^
*T* = 90.0 (5) K
0.22 × 0.10 × 0.05 mm

#### Data collection

Nonius KappaCCD diffractometer (with an Oxford Cryosystems Cryostream cooler)
Absorption correction: multi-scan (*SCALEPACK*; Otwinowski & Minor, 1997 ▶) *T*_min_ = 0.731, *T*_max_ = 0.928
25916 measured reflections
5982 independent reflections
4610 reflections with *I* > 2σ(*I*)
*R*_int_ = 0.045

#### Refinement

*R*[*F* ^2^ > 2σ(*F* ^2^)] = 0.037
*wR*(*F* ^2^) = 0.076
*S* = 1.02
5982 reflections
199 parameters
H-atom parameters constrained
Δρ_max_ = 0.87 e Å^−3^
Δρ_min_ = −1.12 e Å^−3^

### 

Data collection: *COLLECT* (Nonius, 2000 ▶); cell refinement: *SCALEPACK* (Otwinowski & Minor, 1997 ▶); data reduction: *DENZO* (Otwinowski & Minor, 1997 ▶) and *SCALEPACK*; program(s) used to solve structure: *SIR97* (Altomare *et al.*, 1999 ▶); program(s) used to refine structure: *SHELXL97* (Sheldrick, 2008 ▶); molecular graphics: *ORTEP-3 for Windows* (Farrugia, 1997 ▶); software used to prepare material for publication: *SHELXL97*.

## Supplementary Material

Crystal structure: contains datablocks global, I. DOI: 10.1107/S1600536809000439/pv2128sup1.cif

Structure factors: contains datablocks I. DOI: 10.1107/S1600536809000439/pv2128Isup2.hkl

Additional supplementary materials:  crystallographic information; 3D view; checkCIF report

## Figures and Tables

**Table 1:** Hydrogen-bond geometry (Å, °)

*D*—H⋯*A*	*D*—H	H⋯*A*	*D*⋯*A*	*D*—H⋯*A*
O4—H41⋯O1^i^	0.80	1.99	2.785 (3)	169
O4—H42⋯Cl2^ii^	0.80	2.37	3.116 (2)	156

Symmetry codes: (i) 
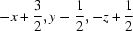
; (ii) 
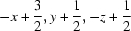
.

**Fig. 1.**
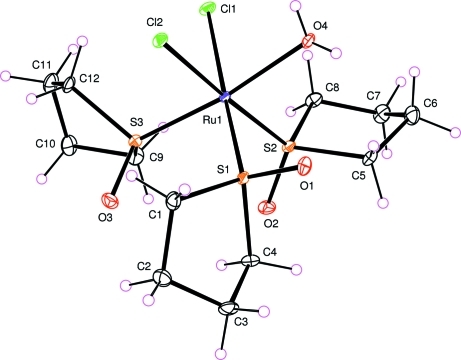
A plot of the title compound with displacement ellipsoids drawn at the 50% level and H atoms having arbitrary radius.

**Fig. 2.**
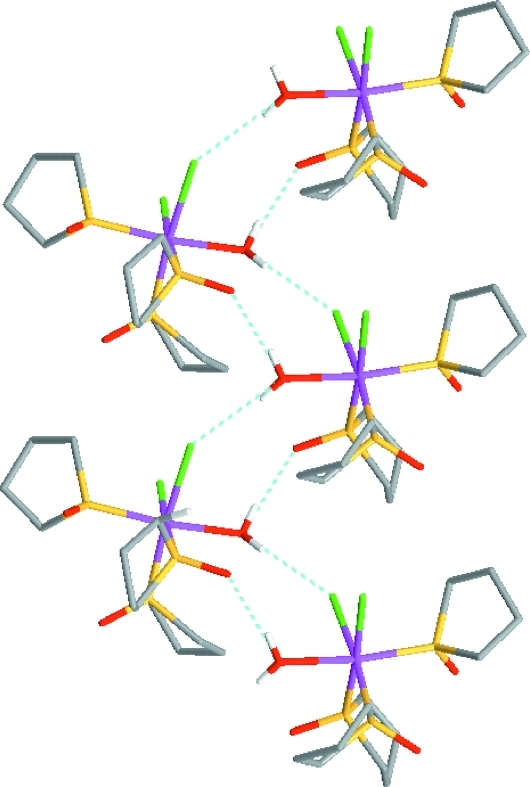
A portion of a hydrogen-bonded chain in the [010] direction.

**Fig. 3.**
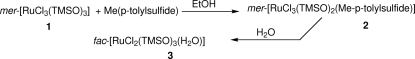
The formation of the title compound.

## supplementary crystallographic information

### Comment

During the course of our studies on ruthenium-DMSO/TMSO complexes, *mer*-RuCl_3_(TMSO)_3_ was refluxed with methyl-*p*-tolylsulfide in absolute ethanol for 1 h. In view of anticancer properties of Ru-DMSO/TMSO complexes, we envision to interact *mer*-RuCl_3_(TMSO)_3_, (1) with sulfur donor ligands, because sulfur-containing ligands are able to bind the metal center strongly and prevent interactions with sulfur-containing enzymes (Aldinucci *et al.*, 2007). In fact, these reactions are believed to be responsible for the nephrotoxicity induced by the platinum (II)-based drugs. However, the compound (2) was hydrolyzed on long standing in solution, and finally the title compound, *fac*-[RuCl_2_(TMSO)_3_(H_2_O)] (3) was isolated. A plausible mechanism of the formation of (3) is shown in scheme 2.

There are three geometrical isomers of the title compound: *trans*,*mer*; *cis*,*mer*; and *fac*. The reported structure is found to be the latter, with chloro groups *cis* and TMSO groups facial, as shown in Fig. 1. More isomers are possible, considering that the TMSO ligands may be coordinated to Ru through either S or O in the same complex, (Srivastava & Fronczek, 2003; Srivastava *et al.*, 2004); however, all are S-coordinated here. Relevant bond distances are given in the supplementary Tables. Most noteworthy is that the Ru—S3 distance, *trans* to water, is 0.03–0.04 Å shorter than the two Ru—S distances *trans* to Cl. While a search of the Cambridge Structural Database (version 5.29, Jan. 2008; Allen, 2002) for Ru complexes with S-bonded TMSO *trans* to water produced no hits, eleven examples of such DMSO complexes were found, refcodes AQAXIZ, AQAXOF, AQAXUL, BINBAC, CECSUZ, QUDRUC, TEXMOZ, TEXNEQ, TEXNIU, WOHNEM, AND WOHNIQ. Those have mean Ru—O distance 2.142 Å and mean Ru—S distance 2.256 Å. Our Ru—O distance, 2.1540 (19) Å, is near the high end of that sample, and our Ru—S distance, Ru1 S3 2.2254 (8) Å, is shorter than any in that sample.

The coordinated water molecule donates an intermolecular hydrogen bond to sulfoxide O and another to Cl, on two different molecules related by unit translation in the b direction. Thus, rings of graph set (Etter, 1990) symbol *R*^2^_2_(9) form chains along [010], propagated by the 2_1_ axis, as shown in Fig. 2.

### Experimental

*mer*-RuCl_3_(TMSO)_3_ (0.166 g, 0.233 mmol) was refluxed with methyl-*p*-tolylsulfide (73 µl, 0.533 mmol) in absolute ethanol (15 ml) for 2 h, followed by cooling to room temperature. Upon standing for eight months, colorless needles of the title compound formed.

### Refinement

H atoms on C were placed in idealized positions with C—H distances 0.99 Å and thereafter treated as riding. Water H atoms were located in difference maps, idealized to have O–H distance 0.80 Å, and treated as riding. *U*_iso_ for H was assigned as 1.2 times *U*_eq_ of the attached atoms (1.5 for H_2_O). The largest negative feature in the final difference map was located 0.75 Å from the Ru position.

### Crystal data

**Table:**

[RuCl_2_(C_4_H_8_OS)_3_(H_2_O)]	*F*(000) = 1024
*M**_r_* = 502.48	*D*_x_ = 1.848 Mg m^−^^3^
Monoclinic, *P*2_1_/*n*	Mo *K*α radiation, λ = 0.71073 Å
Hall symbol: -P 2yn	Cell parameters from 6027 reflections
*a* = 14.302 (3) Å	θ = 2.5–31.5°
*b* = 7.7877 (15) Å	µ = 1.52 mm^−^^1^
*c* = 17.248 (3) Å	*T* = 90 K
β = 109.917 (9)°	Needle, colorless
*V* = 1806.2 (6) Å^3^	0.22 × 0.10 × 0.05 mm
*Z* = 4	

### Data collection

**Table:**

Nonius KappaCCD diffractometer (with an Oxford Cryosystems Cryostream cooler)	5982 independent reflections
Radiation source: fine-focus sealed tube	4610 reflections with *I* > 2σ(*I*)
graphite	*R*_int_ = 0.045
ω and φ scans	θ_max_ = 31.5°, θ_min_ = 2.9°
Absorption correction: multi-scan (*SCALEPACK*; Otwinowski & Minor, 1997)	*h* = −20→21
*T*_min_ = 0.731, *T*_max_ = 0.928	*k* = −11→11
25916 measured reflections	*l* = −25→25

### Refinement

**Table:**

Refinement on *F*^2^	Primary atom site location: structure-invariant direct methods
Least-squares matrix: full	Secondary atom site location: difference Fourier map
*R*[*F*^2^ > 2σ(*F*^2^)] = 0.037	Hydrogen site location: inferred from neighbouring sites
*wR*(*F*^2^) = 0.076	H-atom parameters constrained
*S* = 1.02	*w* = 1/[σ^2^(*F*_o_^2^) + (0.0256*P*)^2^ + 2.7566*P*] where *P* = (*F*_o_^2^ + 2*F*_c_^2^)/3
5982 reflections	(Δ/σ)_max_ = 0.002
199 parameters	Δρ_max_ = 0.87 e Å^−^^3^
0 restraints	Δρ_min_ = −1.12 e Å^−^^3^

### Special details

**Table:**

Geometry. All e.s.d.'s (except the e.s.d. in the dihedral angle between two l.s. planes) are estimated using the full covariance matrix. The cell e.s.d.'s are taken into account individually in the estimation of e.s.d.'s in distances, angles and torsion angles; correlations between e.s.d.'s in cell parameters are only used when they are defined by crystal symmetry. An approximate (isotropic) treatment of cell e.s.d.'s is used for estimating e.s.d.'s involving l.s. planes.
Refinement. Refinement of *F*^2^ against ALL reflections. The weighted *R*-factor *wR* and goodness of fit *S* are based on *F*^2^, conventional *R*-factors *R* are based on *F*, with *F* set to zero for negative *F*^2^. The threshold expression of *F*^2^ > σ(*F*^2^) is used only for calculating *R*-factors(gt) *etc*. and is not relevant to the choice of reflections for refinement. *R*-factors based on *F*^2^ are statistically about twice as large as those based on *F*, and *R*- factors based on ALL data will be even larger.

### Fractional atomic coordinates and isotropic or equivalent isotropic displacement parameters (Å^2^)

**Table:**

	*x*	*y*	*z*	*U*_iso_*/*U*_eq_	
Ru1	0.589322 (15)	0.17421 (3)	0.298795 (12)	0.00590 (5)	
Cl1	0.63294 (5)	−0.00620 (8)	0.41988 (4)	0.01063 (12)	
Cl2	0.59746 (5)	−0.07251 (8)	0.21491 (4)	0.00956 (12)	
S1	0.55748 (5)	0.33080 (8)	0.18270 (4)	0.00718 (12)	
S2	0.59211 (5)	0.41572 (8)	0.37280 (4)	0.00785 (12)	
S3	0.42982 (5)	0.11754 (8)	0.27675 (4)	0.00811 (12)	
O1	0.64973 (14)	0.3915 (2)	0.16851 (11)	0.0112 (4)	
O2	0.49707 (14)	0.5110 (2)	0.35546 (12)	0.0125 (4)	
O3	0.35604 (14)	0.1775 (3)	0.19762 (12)	0.0133 (4)	
O4	0.74739 (13)	0.1973 (2)	0.32611 (11)	0.0094 (4)	
H41	0.7697	0.1034	0.3254	0.014*	
H42	0.7735	0.2581	0.3021	0.014*	
C1	0.4799 (2)	0.2324 (3)	0.08568 (16)	0.0117 (5)	
H1A	0.5196	0.2111	0.0495	0.014*	
H1B	0.4527	0.1216	0.0965	0.014*	
C2	0.3955 (2)	0.3583 (4)	0.04466 (17)	0.0142 (6)	
H2A	0.3353	0.3260	0.0573	0.017*	
H2B	0.3790	0.3561	−0.0159	0.017*	
C3	0.4301 (2)	0.5380 (4)	0.07805 (16)	0.0129 (5)	
H3A	0.4794	0.5831	0.0545	0.016*	
H3B	0.3731	0.6183	0.0645	0.016*	
C4	0.4770 (2)	0.5151 (3)	0.17114 (16)	0.0109 (5)	
H4A	0.4256	0.4934	0.1964	0.013*	
H4B	0.5157	0.6181	0.1970	0.013*	
C5	0.6859 (2)	0.5663 (3)	0.36345 (16)	0.0108 (5)	
H5A	0.7141	0.5249	0.3218	0.013*	
H5B	0.6559	0.6808	0.3463	0.013*	
C6	0.7667 (2)	0.5771 (4)	0.44766 (17)	0.0152 (6)	
H6A	0.8007	0.6897	0.4548	0.018*	
H6B	0.8167	0.4854	0.4538	0.018*	
C7	0.7149 (2)	0.5548 (4)	0.51139 (17)	0.0145 (6)	
H7A	0.7644	0.5366	0.5671	0.017*	
H7B	0.6748	0.6576	0.5128	0.017*	
C8	0.6484 (2)	0.3978 (3)	0.48391 (16)	0.0110 (5)	
H8A	0.5967	0.3964	0.5101	0.013*	
H8B	0.6881	0.2911	0.4990	0.013*	
C9	0.3821 (2)	0.1780 (4)	0.35774 (17)	0.0132 (5)	
H9A	0.4359	0.1791	0.4121	0.016*	
H9B	0.3507	0.2928	0.3471	0.016*	
C10	0.3059 (2)	0.0401 (4)	0.35450 (18)	0.0148 (6)	
H10A	0.2462	0.0530	0.3046	0.018*	
H10B	0.2856	0.0455	0.4039	0.018*	
C11	0.3595 (2)	−0.1292 (4)	0.35214 (18)	0.0149 (6)	
H11A	0.4113	−0.1516	0.4063	0.018*	
H11B	0.3117	−0.2258	0.3390	0.018*	
C12	0.4068 (2)	−0.1113 (3)	0.28551 (18)	0.0119 (5)	
H12A	0.3614	−0.1562	0.2323	0.014*	
H12B	0.4699	−0.1764	0.3010	0.014*	

### Atomic displacement parameters (Å^2^)

**Table:**

	*U*^11^	*U*^22^	*U*^33^	*U*^12^	*U*^13^	*U*^23^
Ru1	0.00693 (9)	0.00402 (9)	0.00659 (9)	0.00017 (7)	0.00210 (7)	0.00032 (7)
Cl1	0.0146 (3)	0.0069 (3)	0.0095 (3)	0.0008 (2)	0.0030 (2)	0.0025 (2)
Cl2	0.0109 (3)	0.0061 (3)	0.0119 (3)	0.0004 (2)	0.0042 (2)	−0.0016 (2)
S1	0.0081 (3)	0.0058 (3)	0.0071 (3)	−0.0008 (2)	0.0020 (2)	0.0000 (2)
S2	0.0099 (3)	0.0056 (3)	0.0077 (3)	0.0003 (2)	0.0026 (2)	0.0005 (2)
S3	0.0086 (3)	0.0069 (3)	0.0087 (3)	0.0000 (2)	0.0028 (2)	0.0008 (2)
O1	0.0116 (9)	0.0101 (9)	0.0133 (9)	−0.0033 (7)	0.0060 (8)	0.0002 (7)
O2	0.0117 (9)	0.0097 (9)	0.0171 (10)	0.0036 (7)	0.0060 (8)	0.0003 (8)
O3	0.0093 (9)	0.0158 (9)	0.0127 (9)	−0.0009 (8)	0.0009 (7)	0.0055 (8)
O4	0.0108 (9)	0.0049 (8)	0.0132 (9)	−0.0012 (7)	0.0049 (7)	−0.0001 (7)
C1	0.0127 (13)	0.0110 (12)	0.0102 (12)	−0.0029 (10)	0.0024 (10)	−0.0015 (10)
C2	0.0131 (13)	0.0156 (14)	0.0110 (13)	−0.0005 (10)	0.0005 (10)	0.0025 (10)
C3	0.0146 (13)	0.0125 (12)	0.0113 (13)	0.0028 (11)	0.0038 (10)	0.0071 (10)
C4	0.0115 (12)	0.0086 (12)	0.0121 (13)	0.0040 (10)	0.0034 (10)	0.0024 (10)
C5	0.0143 (13)	0.0061 (11)	0.0123 (12)	−0.0014 (10)	0.0052 (10)	0.0009 (10)
C6	0.0160 (14)	0.0133 (13)	0.0135 (13)	−0.0039 (11)	0.0014 (11)	−0.0030 (11)
C7	0.0197 (15)	0.0130 (13)	0.0083 (12)	−0.0027 (11)	0.0014 (11)	−0.0033 (10)
C8	0.0161 (13)	0.0092 (12)	0.0080 (12)	0.0007 (10)	0.0044 (10)	0.0006 (10)
C9	0.0135 (13)	0.0131 (12)	0.0159 (13)	−0.0003 (11)	0.0086 (11)	−0.0031 (11)
C10	0.0138 (13)	0.0178 (14)	0.0156 (14)	−0.0047 (11)	0.0087 (11)	0.0012 (11)
C11	0.0180 (14)	0.0121 (12)	0.0156 (14)	−0.0055 (11)	0.0068 (11)	0.0024 (11)
C12	0.0107 (12)	0.0063 (11)	0.0199 (14)	−0.0030 (10)	0.0067 (11)	0.0009 (10)

### Geometric parameters (Å, °)

**Table:**

Ru1—O4	2.1540 (19)	C3—H3B	0.9900
Ru1—S3	2.2254 (8)	C4—H4A	0.9900
Ru1—S1	2.2546 (7)	C4—H4B	0.9900
Ru1—S2	2.2657 (7)	C5—C6	1.519 (4)
Ru1—Cl1	2.4161 (7)	C5—H5A	0.9900
Ru1—Cl2	2.4317 (7)	C5—H5B	0.9900
S1—O1	1.498 (2)	C6—C7	1.531 (4)
S1—C4	1.807 (3)	C6—H6A	0.9900
S1—C1	1.831 (3)	C6—H6B	0.9900
S2—O2	1.487 (2)	C7—C8	1.521 (4)
S2—C8	1.814 (3)	C7—H7A	0.9900
S2—C5	1.830 (3)	C7—H7B	0.9900
S3—O3	1.487 (2)	C8—H8A	0.9900
S3—C9	1.813 (3)	C8—H8B	0.9900
S3—C12	1.828 (3)	C9—C10	1.517 (4)
O4—H41	0.8000	C9—H9A	0.9900
O4—H42	0.8000	C9—H9B	0.9900
C1—C2	1.529 (4)	C10—C11	1.532 (4)
C1—H1A	0.9900	C10—H10A	0.9900
C1—H1B	0.9900	C10—H10B	0.9900
C2—C3	1.530 (4)	C11—C12	1.525 (4)
C2—H2A	0.9900	C11—H11A	0.9900
C2—H2B	0.9900	C11—H11B	0.9900
C3—C4	1.525 (4)	C12—H12A	0.9900
C3—H3A	0.9900	C12—H12B	0.9900
			
O4—Ru1—S3	172.92 (5)	C3—C4—S1	104.15 (18)
O4—Ru1—S1	91.63 (5)	C3—C4—H4A	110.9
S3—Ru1—S1	94.04 (3)	S1—C4—H4A	110.9
O4—Ru1—S2	89.52 (5)	C3—C4—H4B	110.9
S3—Ru1—S2	94.66 (3)	S1—C4—H4B	110.9
S1—Ru1—S2	90.62 (3)	H4A—C4—H4B	108.9
O4—Ru1—Cl1	85.23 (5)	C6—C5—S2	107.04 (18)
S3—Ru1—Cl1	88.86 (3)	C6—C5—H5A	110.3
S1—Ru1—Cl1	175.45 (2)	S2—C5—H5A	110.3
S2—Ru1—Cl1	92.63 (3)	C6—C5—H5B	110.3
O4—Ru1—Cl2	86.37 (5)	S2—C5—H5B	110.3
S3—Ru1—Cl2	89.75 (2)	H5A—C5—H5B	108.6
S1—Ru1—Cl2	86.28 (3)	C5—C6—C7	106.5 (2)
S2—Ru1—Cl2	174.78 (2)	C5—C6—H6A	110.4
Cl1—Ru1—Cl2	90.24 (3)	C7—C6—H6A	110.4
O1—S1—C4	107.13 (12)	C5—C6—H6B	110.4
O1—S1—C1	106.01 (12)	C7—C6—H6B	110.4
C4—S1—C1	93.82 (12)	H6A—C6—H6B	108.6
O1—S1—Ru1	113.14 (8)	C8—C7—C6	105.8 (2)
C4—S1—Ru1	117.12 (9)	C8—C7—H7A	110.6
C1—S1—Ru1	117.49 (9)	C6—C7—H7A	110.6
O2—S2—C8	107.31 (12)	C8—C7—H7B	110.6
O2—S2—C5	108.06 (12)	C6—C7—H7B	110.6
C8—S2—C5	93.85 (12)	H7A—C7—H7B	108.7
O2—S2—Ru1	117.48 (8)	C7—C8—S2	105.84 (18)
C8—S2—Ru1	116.59 (9)	C7—C8—H8A	110.6
C5—S2—Ru1	110.80 (9)	S2—C8—H8A	110.6
O3—S3—C9	106.85 (13)	C7—C8—H8B	110.6
O3—S3—C12	106.88 (12)	S2—C8—H8B	110.6
C9—S3—C12	93.64 (13)	H8A—C8—H8B	108.7
O3—S3—Ru1	117.39 (8)	C10—C9—S3	104.01 (19)
C9—S3—Ru1	116.73 (10)	C10—C9—H9A	111.0
C12—S3—Ru1	112.49 (9)	S3—C9—H9A	111.0
Ru1—O4—H41	108.3	C10—C9—H9B	111.0
Ru1—O4—H42	125.1	S3—C9—H9B	111.0
H41—O4—H42	105.9	H9A—C9—H9B	109.0
C2—C1—S1	106.96 (18)	C9—C10—C11	104.5 (2)
C2—C1—H1A	110.3	C9—C10—H10A	110.9
S1—C1—H1A	110.3	C11—C10—H10A	110.9
C2—C1—H1B	110.3	C9—C10—H10B	110.9
S1—C1—H1B	110.3	C11—C10—H10B	110.9
H1A—C1—H1B	108.6	H10A—C10—H10B	108.9
C1—C2—C3	108.0 (2)	C12—C11—C10	107.2 (2)
C1—C2—H2A	110.1	C12—C11—H11A	110.3
C3—C2—H2A	110.1	C10—C11—H11A	110.3
C1—C2—H2B	110.1	C12—C11—H11B	110.3
C3—C2—H2B	110.1	C10—C11—H11B	110.3
H2A—C2—H2B	108.4	H11A—C11—H11B	108.5
C4—C3—C2	105.0 (2)	C11—C12—S3	106.86 (19)
C4—C3—H3A	110.7	C11—C12—H12A	110.4
C2—C3—H3A	110.7	S3—C12—H12A	110.4
C4—C3—H3B	110.7	C11—C12—H12B	110.4
C2—C3—H3B	110.7	S3—C12—H12B	110.4
H3A—C3—H3B	108.8	H12A—C12—H12B	108.6
			
O4—Ru1—S1—O1	−0.88 (10)	S1—Ru1—S3—C12	129.51 (10)
S3—Ru1—S1—O1	−176.63 (9)	S2—Ru1—S3—C12	−139.54 (10)
S2—Ru1—S1—O1	88.66 (9)	Cl1—Ru1—S3—C12	−46.99 (10)
Cl2—Ru1—S1—O1	−87.14 (9)	Cl2—Ru1—S3—C12	43.26 (10)
O4—Ru1—S1—C4	−126.24 (11)	O1—S1—C1—C2	−105.28 (19)
S3—Ru1—S1—C4	58.01 (11)	C4—S1—C1—C2	3.8 (2)
S2—Ru1—S1—C4	−36.70 (11)	Ru1—S1—C1—C2	127.11 (16)
Cl2—Ru1—S1—C4	147.50 (11)	S1—C1—C2—C3	23.5 (3)
O4—Ru1—S1—C1	123.22 (11)	C1—C2—C3—C4	−46.0 (3)
S3—Ru1—S1—C1	−52.53 (11)	C2—C3—C4—S1	47.2 (2)
S2—Ru1—S1—C1	−147.24 (10)	O1—S1—C4—C3	78.5 (2)
Cl2—Ru1—S1—C1	36.96 (10)	C1—S1—C4—C3	−29.5 (2)
O4—Ru1—S2—O2	158.55 (10)	Ru1—S1—C4—C3	−153.17 (15)
S3—Ru1—S2—O2	−27.18 (9)	O2—S2—C5—C6	117.47 (19)
S1—Ru1—S2—O2	66.92 (9)	C8—S2—C5—C6	7.9 (2)
Cl1—Ru1—S2—O2	−116.25 (9)	Ru1—S2—C5—C6	−112.51 (17)
O4—Ru1—S2—C8	−72.00 (12)	S2—C5—C6—C7	−33.1 (3)
S3—Ru1—S2—C8	102.28 (11)	C5—C6—C7—C8	48.6 (3)
S1—Ru1—S2—C8	−163.62 (11)	C6—C7—C8—S2	−41.6 (3)
Cl1—Ru1—S2—C8	13.20 (11)	O2—S2—C8—C7	−90.7 (2)
O4—Ru1—S2—C5	33.70 (11)	C5—S2—C8—C7	19.5 (2)
S3—Ru1—S2—C5	−152.03 (9)	Ru1—S2—C8—C7	135.11 (16)
S1—Ru1—S2—C5	−57.93 (10)	O3—S3—C9—C10	80.2 (2)
Cl1—Ru1—S2—C5	118.90 (10)	C12—S3—C9—C10	−28.6 (2)
S1—Ru1—S3—O3	4.88 (10)	Ru1—S3—C9—C10	−146.10 (16)
S2—Ru1—S3—O3	95.83 (10)	S3—C9—C10—C11	48.1 (2)
Cl1—Ru1—S3—O3	−171.62 (10)	C9—C10—C11—C12	−48.9 (3)
Cl2—Ru1—S3—O3	−81.37 (10)	C10—C11—C12—S3	26.8 (3)
S1—Ru1—S3—C9	−123.92 (11)	O3—S3—C12—C11	−107.7 (2)
S2—Ru1—S3—C9	−32.96 (11)	C9—S3—C12—C11	1.2 (2)
Cl1—Ru1—S3—C9	59.59 (11)	Ru1—S3—C12—C11	122.10 (17)
Cl2—Ru1—S3—C9	149.83 (11)		

### Hydrogen-bond geometry (Å, °)

**Table:**

*D*—H···*A*	*D*—H	H···*A*	*D*···*A*	*D*—H···*A*
O4—H41···O1^i^	0.80	1.99	2.785 (3)	169
O4—H42···Cl2^ii^	0.80	2.37	3.116 (2)	156

Symmetry codes: (i) −*x*+3/2, *y*−1/2, −*z*+1/2; (ii) −*x*+3/2, *y*+1/2, −*z*+1/2.
